# Paraneoplastic neuromyelitis optica spectrum disorder with dual AQP4-IgG and CRMP5 antibodies following thymectomy: a case report

**DOI:** 10.3389/fnins.2026.1747586

**Published:** 2026-03-09

**Authors:** Yucai Chuai, Tao Jin, Lizhi Zhang, Honglu Song

**Affiliations:** 1 Department of Special Medical Services, Bethune International Peace Hospital, Shijiazhuang, Hebei, China; 2 Department of Ophthalmology, Bethune International Peace Hospital, Shijiazhuang, Hebei, China

**Keywords:** aquaporin-4, CRMP5, neuromyelitis optica spectrum disorder, paraneoplastic syndrome, thymoma

## Abstract

We present a rare case of paraneoplastic neuromyelitis optica spectrum disorder (NMOSD) in a 50-year-old woman with a history of B3 thymoma, marked by dual positivity for AQP4-IgG and CV2/CRMP5 antibodies. The patient developed sequential bilateral optic neuritis, with orbital MRI revealing enhancement of the left optic nerve. Serological analysis showed a high AQP4-IgG titer (1:320) and positive CV2/CRMP5 antibodies, while other paraneoplastic markers were absent. Treatment with glucocorticoids and mycophenolate mofetil led to substantial visual recovery. This case underscores the association between thymoma and paraneoplastic NMOSD and suggests a potential interaction between AQP4- and CRMP5-related autoimmune responses. Although CV2/CRMP5 antibodies are typically linked to lung cancer and thymoma, their coexistence with AQP4-IgG in NMOSD points to a distinct paraneoplastic mechanism. These findings support the need for thorough tumor screening in NMOSD patients with atypical features, particularly those with CV2/CRMP5 seropositivity.

## Introduction

Neuromyelitis optica spectrum disorder (NMOSD) is a rare autoimmune demyelinating condition of the central nervous system, predominantly affecting the optic nerves and spinal cord. In most cases, it is associated with pathogenic IgG antibodies targeting aquaporin-4 (AQP4) (Huda et al., 2019). Recent studies indicate that 3–25% of NMOSD cases may be paraneoplastic, with malignancy-driven immune responses triggering neurological symptoms either before or after cancer diagnosis (Virgilio et al., 2021). This immunopathogenesis parallels that of CRMP5 antibody-associated paraneoplastic neurological syndromes (PNS), often linked to lung cancer and thymoma (Graus and Dalmau, 2012). We describe a rare case of NMOSD with dual positivity for AQP4-IgG and CRMP5 antibodies following thymectomy, suggesting an overlap between NMOSD and PNS autoimmunity. This co-occurrence points to possible shared mechanisms, particularly in thymoma-related immune dysregulation. The case highlights the need for comprehensive cancer screening in seropositive NMOSD patients, especially those with atypical features or coexisting paraneoplastic antibodies, to support early diagnosis and guide immunotherapy.

## Case report

A 50-year-old woman with a history of thymectomy was admitted for progressive bilateral vision loss, reporting a 4-month decline in the right eye and a 2-week history of left eye involvement. Three years earlier, she presented with diplopia and right eyelid ptosis, leading to a diagnosis of myasthenia gravis. Chest CT at that time revealed a suspected invasive thymoma in the left mid-upper mediastinum. She underwent complete surgical resection at a local hospital, and histopathology confirmed a B3-type thymoma in the anterior mediastinum. Immunohistochemistry showed epithelial marker positivity (PCK+, EMA+), absence of CD117, and lymphoid marker positivity for CD1a, TdT, CD99, and CD5. The Ki-67 index was 20%. Postoperatively, she received 33 sessions of radiotherapy and five cycles of chemotherapy, after which pyridostigmine bromide was discontinued.

Four months prior to the current admission, she developed right eye pain and visual blurring, progressing to complete loss of light perception within 4 weeks. A post-thymectomy PET-CT with seed implantation revealed mild metabolic heterogeneity at the surgical site, with no evidence of pulmonary or distant metastases. She received intravenous methylprednisolone (1 g/day for 3 days), followed by oral prednisone (60 mg/day), resulting in partial visual improvement to counting fingers in the right eye. However, all medications were self-discontinued 5 days after discharge without medical consultation.

**Figure 1:**
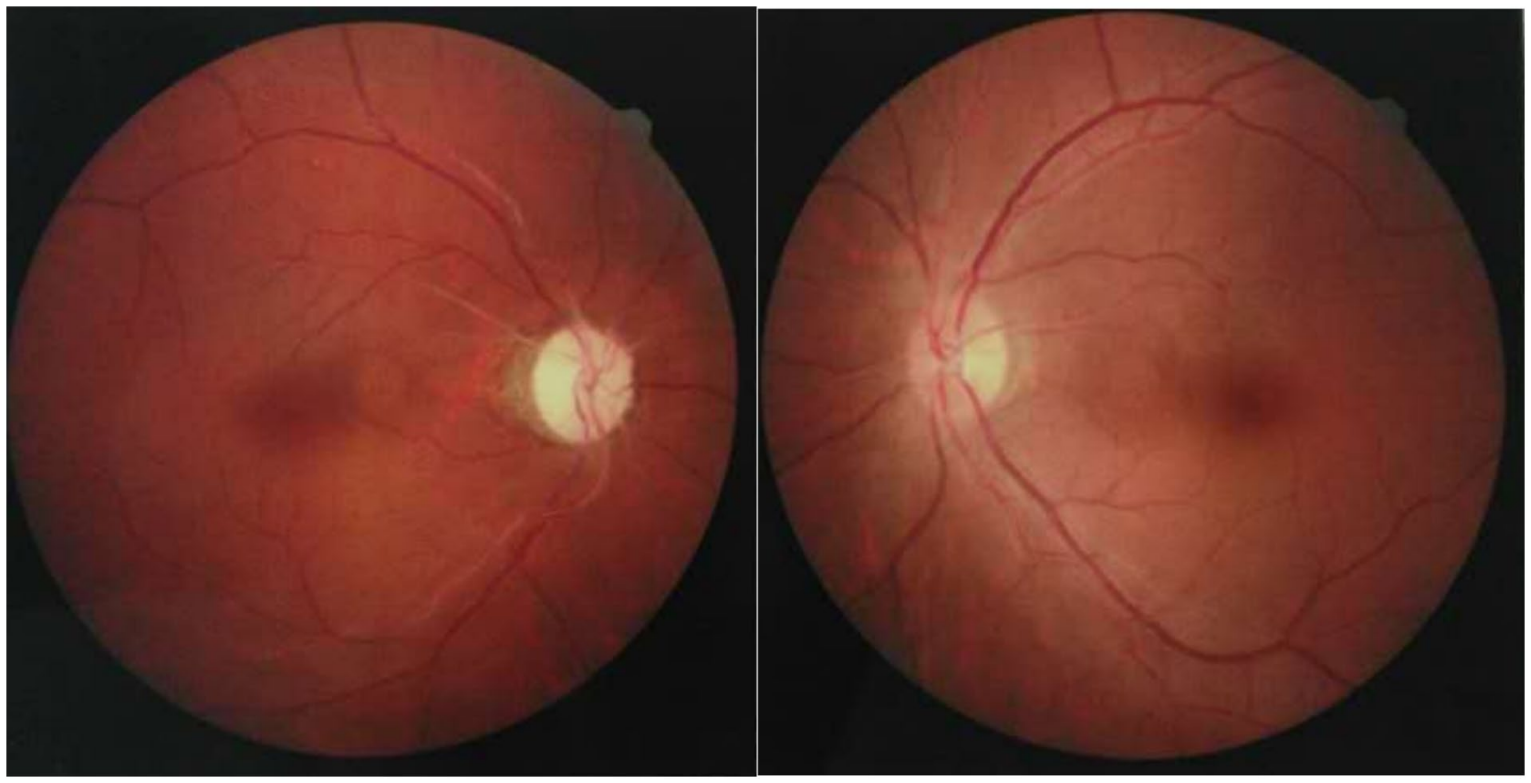
Right optic disc atrophy and mild edema of the left optic disc.

**Figure 2:**
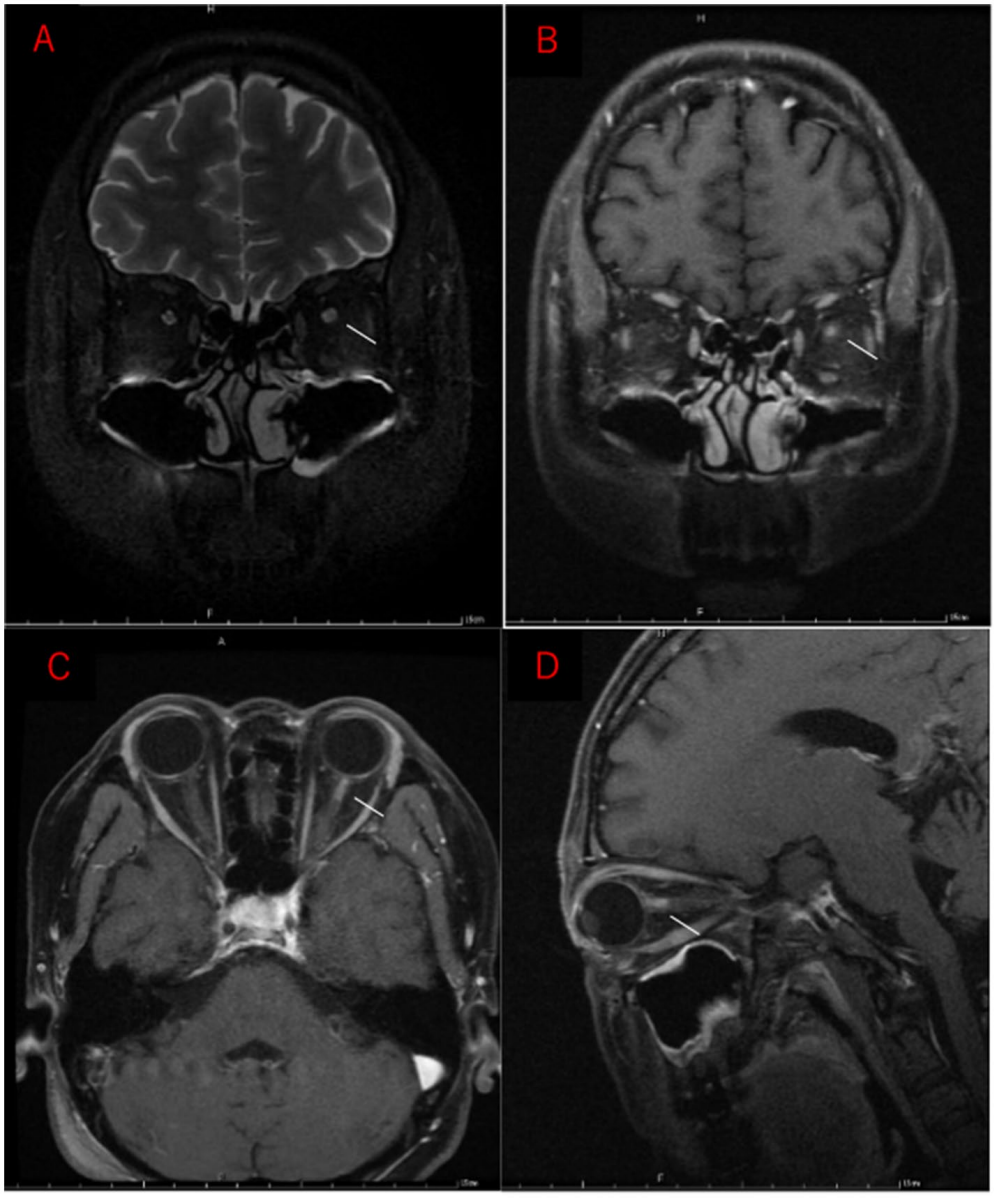
Orbital MRI performed 2 weeks after onset of left-eye vision loss. **(A)** Coronal T2-weighted image shows hyperintensity in the anterior intraorbital segment of the left optic nerve. **(B–D)** Post-contrast T1-weighted images demonstrate corresponding enhancement of the affected segment (white arrow).

Two weeks following an upper respiratory infection, the patient experienced acute visual loss in the left eye, accompanied by periorbital pain and headache. Vision rapidly declined to no light perception within 72 h. Ophthalmologic examination revealed no signs of anterior segment inflammation, including absence of conjunctival injection, keratic precipitates, or anterior chamber cells in either eye. Fundoscopy (Figure 1) revealed optic disc edema in the left eye and optic atrophy in the right. Orbital MRI (Figure 2) demonstrated typical inflammatory findings: coronal T2-weighted imaging showed hyperintensity in the anterior intraorbital segment of the left optic nerve, with corresponding enhancement on post-contrast T1-weighted imaging. Brain MRI with contrast revealed no parenchymal or leptomeningeal metastases.

Serologic testing revealed high-titer AQP4-IgG positivity (1:320, cell-based assay; Euroimmun). AQP4-IgG was also present in the cerebrospinal fluid (CSF) at a titer of 1:3.2. Paraneoplastic antibody screening was negative for anti-Hu, Ri, Yo, and amphiphysin, but strongly positive for anti-CV2/CRMP5. CSF analysis showed an acellular profile (2 WBCs/μL) with normal protein (293.3 mg/L) and glucose (3.7 mmol/L). Furthermore, CSF cytology was negative for malignant cells, supporting a non-neoplastic, autoimmune etiology. Additional testing showed mildly elevated anti-β2 glycoprotein I antibodies (61.64 RU/mL), with normal anti-cardiolipin levels (19.29 RU/mL). Tests for infectious and other autoimmune markers, including acetylcholine receptor antibodies, were unremarkable.

The patient was treated with intravenous methylprednisolone (1 g daily for 3 days, followed by 500 mg daily for 3 days), then transitioned to oral prednisone (60 mg daily with tapering). Visual acuity improved significantly, reaching 20/30 in the left eye. Maintenance therapy with mycophenolate mofetil (750 mg twice daily) was initiated to prevent relapse. At six-month follow-up, the patient remained stable without recurrence of optic neuritis, indicating durable efficacy of the combined mycophenolate and low-dose prednisone regimen.

## Discussion

This rare case of AQP4-IgG-positive NMOSD with coexisting CV2/CRMP5 antibodies broadens the current understanding of paraneoplastic autoimmunity. The patient’s history of thymoma and sequential bilateral optic neuritis points to two distinct yet potentially interacting autoimmune mechanisms. CRMP5 antibodies, first identified by Honnorat et al. (1996) as markers of oligodendrocyte-related pathology, are most often associated with small-cell lung cancer and thymoma (Monstad et al., 2008). Their presence here supports a paraneoplastic origin of the neurological symptoms, despite thymectomy occurring 3 years earlier. The thymus plays a central role in T-cell maturation and immune tolerance. Surgical removal may disrupt these regulatory pathways, promoting the loss of self-tolerance and subsequent autoantibody production. This dysregulation could plausibly lead to the concurrent generation of AQP4-IgG and CRMP5 antibodies, targeting distinct yet overlapping CNS antigens.

The detection of AQP4-IgG introduces further immunopathogenic implications. AQP4 is expressed in both astrocytes and thymic epithelial cells (Chan et al., 2010) and may become immunogenic following thymoma-associated immune tolerance breakdown. Approximately 5% of AQP4-IgG-positive cases are linked to malignancy (Pittock and Lennon, 2008). In this patient, a high AQP4-IgG titer (1:320) and rapid steroid response favor an autoimmune mechanism driven by prior thymoma rather than direct neoplastic involvement—consistent with reports of favorable immunotherapy responses in paraneoplastic NMOSD (Sepúlveda et al., 2018).

The timing of symptom onset following thymoma treatment is notable. The initial diagnosis of myasthenia gravis, followed 3 years later by optic neuritis after tumor resection and radiotherapy, parallels the “dual-hit” phenomenon seen in about 12% of thymoma-related myasthenia gravis cases with CRMP5 antibodies (Monstad et al., 2008). This pattern suggests that tumor removal may unmask latent autoimmunity by disturbing immune homeostasis. The patient’s visual improvement with corticosteroids, followed by relapse after self-discontinuation, underscores the need for sustained immunosuppression in paraneoplastic NMOSD (Dubey et al., 2018). The absence of tumor recurrence on PET-CT supports ongoing autoimmune activity as the primary cause of symptoms, rather than occult malignancy.

A critical diagnostic consideration in this case is whether the optic neuritis is primarily attributable to CRMP5 autoimmunity or AQP4-IgG-mediated NMOSD. CRMP5-related optic neuritis, although rare, is a well-established entity often presenting with chronic, painless visual loss and occasionally concurrent retinitis (Dubey et al., 2018). In contrast, AQP4-IgG-related optic neuritis typically presents with acute, severe, and painful vision loss, frequently involving the optic chiasm and long-segment optic nerve (>1/2 of the optic nerve length) on MRI. In our patient, the acute onset with periorbital pain, rapid progression to no light perception within 72 h, and high-titer AQP4-IgG seropositivity strongly favor AQP4-IgG-mediated pathology. We acknowledge that the MRI finding of relatively short-segment enhancement without chiasmal involvement is somewhat atypical for classical NMOSD. However, recent evidence suggests that paraneoplastic NMOSD may exhibit atypical MRI features, and the absence of chiasmal involvement does not exclude the diagnosis (Sepúlveda et al., 2018). We hypothesize that this case represents a pathogenic overlap: the clinical phenotype is driven by AQP4-IgG, while CRMP5 serves as a serological footprint of the underlying thymoma-induced breakdown of immune tolerance.

This case presents several key diagnostic insights. While CV2/CRMP5 antibodies are typically associated with encephalomyelitis or peripheral neuropathy, their detection in isolated optic neuritis expands the known clinical spectrum. In Yu et al.’s (2001) study, only 7% of CRMP5-positive patients exhibited optic neuritis, making this presentation especially rare—particularly alongside AQP4-IgG positivity. The PNS-Care criteria classify CV2/CRMP5 as high-risk paraneoplastic antibodies (>80% tumor association) (Graus et al., 2021), yet the paraneoplastic potential of AQP4-IgG remains underrecognized. This patient met the criteria for a definitive PNS based on: (1) a classical syndrome (optic neuritis), (2) a high-risk antibody (CRMP5), and (3) a confirmed thymoma within 5 years. This triad underscores the importance of broad antibody screening in atypical NMOSD, particularly in patients with red flags such as age over 50—where malignancy risk exceeds 20%—or a cancer history (Ding et al., 2021; Beauchemin et al., 2018).

To assist clinicians in recognizing and managing similar cases, we summarize the characteristic features of paraneoplastic NMOSD based on the current literature (Virgilio et al., 2021; Sepúlveda et al., 2018; Ding et al., 2021). Associated tumors include thymoma, lung cancer (particularly small cell lung cancer), breast cancer, and ovarian teratoma. Clinical phenotypes are dominated by optic neuritis (often bilateral or sequential) and longitudinally extensive transverse myelitis, although area postrema syndrome and brainstem encephalitis have also been reported. CSF findings are typically non-specific, with mild pleocytosis or normal cell counts, and importantly, absence of malignant cells in immune-mediated cases. Concomitant paraneoplastic antibodies beyond AQP4-IgG may include anti-CRMP5, Hu, Ri, Yo, and amphiphysin, each suggesting distinct tumor associations and neurological presentations. The recognition of these features—particularly when NMOSD presents at an older age, follows a tumor diagnosis, or is accompanied by atypical antibodies—should prompt comprehensive oncological evaluation and multidisciplinary management.

Management in such cases remains challenging. Although high-dose corticosteroids elicited a rapid response, long-term immunosuppression strategies remain uncertain. Cai et al. (2016) reported favorable outcomes in thymoma-associated paraneoplastic NMOSD, but extended immunotherapy poses risks, including secondary malignancies. Tumor-directed treatment may help modulate autoimmunity, as shown in ovarian teratoma-associated NMOSD where AQP4-IgG titers declined after tumor resection (Liu et al., 2024). However, in this case, prior thymectomy did not prevent subsequent CNS autoimmunity, suggesting persistent—and potentially irreversible—immune dysregulation.

## Conclusion

This case presents a rare overlap of AQP4-IgG-positive NMOSD and CV2/CRMP5 antibodies in a thymoma patient, emphasizing the paraneoplastic contribution to autoimmune demyelination. In atypical NMOSD, early cancer screening and coordinated multidisciplinary care are critical. Dual antibody positivity points to complex immune cross-reactivity, warranting further investigation into underlying mechanisms and therapeutic strategies.

## Data Availability

The original contributions presented in the study are included in the article/supplementary material, further inquiries can be directed to the corresponding author.
